# Human cell-derived tissue-engineered heart valve with integrated Valsalva sinuses: towards native-like transcatheter pulmonary valve replacements

**DOI:** 10.1038/s41536-019-0077-4

**Published:** 2019-06-17

**Authors:** Sarah E. Motta, Valentina Lintas, Emanuela S. Fioretta, Petra E. Dijkman, Matilde Putti, Etem Caliskan, Héctor Rodriguez Cetina Biefer, Miriam Lipiski, Mareike Sauer, Nikola Cesarovic, Simon P. Hoerstrup, Maximilian Y. Emmert

**Affiliations:** 1 Institute for Regenerative Medicine (IREM), University of Zurich, Zurich, Switzerland; 2 Department of Biomedical Engineering, Technische Universiteit Eindhoven, Eindhoven, The Netherlands; 3 Department of Cardiovascular Surgery, Charité Universitätsmedizin Berlin, Berlin, Germany; 4 Department of Cardiothoracic and Vascular Surgery, German Heart Center Berlin, Berlin, Germany; 5 Division of Surgical Research, University Hospital Zürich, University of Zurich, Zurich, Switzerland; 6 Wyss Translational Center Zurich, University of Zurich and ETH Zurich, Zurich, Switzerland

**Keywords:** Preclinical research, Translational research, Valvular disease

## Abstract

Transcatheter valve replacement indication is currently being extended to younger and lower-risk patients. However, transcatheter prostheses are still based on glutaraldehyde-fixed xenogeneic materials. Hence, they are prone to calcification and long-term structural degeneration, which are particularly accelerated in younger patients. Tissue-engineered heart valves based on decellularized in vitro grown tissue-engineered matrices (TEM) have been suggested as a valid alternative to currently used bioprostheses, showing good performance and remodeling capacity as transcatheter pulmonary valve replacement (TPVR) in sheep. Here, we first describe the in vitro development of human cell-derived TEM (hTEM) and their application as tissue-engineered sinus valves (hTESVs), endowed with Valsalva sinuses for TPVR. The hTEM and hTESVs were systematically characterized in vitro by histology, immunofluorescence, and biochemical analyses, before they were evaluated in a pulse duplicator system under physiological pulmonary pressure conditions. Thereafter, transapical delivery of hTESVs was tested for feasibility and safety in a translational sheep model, achieving good valve performance and early cellular infiltration. This study demonstrates the principal feasibility of clinically relevant hTEM to manufacture hTESVs for TPVR.

## Introduction

Transcatheter pulmonary valve replacement (TPVR) represents a valid option treatment for patients suffering from valvular heart diseases.^1^ Current clinically approved heart valve substitutes for TPVR consist of glutaraldehyde-fixed animal-derived materials (e.g., bovine and porcine pericardium and porcine heart valves).^2^ Owing to their animal origin, such substitutes are prone to progressive structural degradation and calcific degeneration, thus requiring multiple substitutive re-interventions, which are particularly critical for young patients.^3^ Decellularized human heart valves (allografts) are considered a valid alternative to xenoprostheses, ensuring good performance and reduced reoperation rates.^4^ However, several drawbacks—such as donor shortage, lack of long-term follow-up studies, and inability to grow—still limit the broad clinical adoption of such prostheses.^5^ To solve the rising clinical need for heart valve substitutes, heart valve tissue engineering (TE) has been proposed as a potential solution to generate living native-analogous heart valves with long-term regenerative and remodeling capacities.^6^ Introduced >20 years ago by Langer and Vacanti, cellularized in vitro grown tissue-engineered heart valves (TEHVs) rely on the use of autologous, patient-specific cell sources, to create an immunologically safe product.^7^ However, this approach has also severe limitations by being non-scalable, not suitable for emergency, time consuming, and affected by patient-to-patient variability in the final product.^8–12^ Hence, a way to reduce the logistical complexity and prosthesis variability was identified in the use of cell-free constructs with the potential to remodel and regenerate upon implantation.^6^ This led to the development of novel in situ TE concepts, that rely on the ability of the recipient’s body to regenerate and remodel the TEHVs once implanted in vivo.^6,13–15^ Decellularization of in vitro grown tissue-engineered matrices (TEM) has been proposed as a possible option to provide TEHVs with in situ regenerative properties and off-the-shelf availability, creating a clinically relevant prosthesis.^16–26^ Decellularized TEM showed efficient cell depletion as well as matrix preservation in a comparative in vitro study,^16^ as well as in several in vivo studies.^16–18,21,22^ Here, decellularized TEM-based TEHVs demonstrated superior long-term functionality and comparable mechanical properties to cellularized in vitro grown TEHVs. In support of this, numerous preclinical studies have proven the regenerative properties of decellularized TEM-based TEHVs, also when implanted with transcatheter techniques in large preclinical animal models.^17–22^ However, independently of the used bioengineering methodology, TEHVs functionality is gradually lost within few months owing to cell-mediated leaflet retraction.^13,17,20,22–24,27^ To solve this problem, computational modeling tools have been recently applied to TEM-based TEHVs^18,28,29^ to investigate the effect of physiological values of stress and strain on leaflet remodeling. These studies suggested that TEHVs with leaflets characterized by a more physiological-like belly curvature showed limited tissue compression, and therefore reduced leaflet retraction during diastole.^18,28,30^ By using an insert to implement such design,^18,28,29^ the TEM-based TEHVs have been implanted in the sheep model,^18^ demonstrating excellent long-term functionality and reduction of the maladaptive remodeling phenomena observed in the previous generations of TEHVs (i.e., leaflet retraction and valve insufficiency).^9,17,21,22^ These results suggest the importance of mimicking the physiological valve design to ensure long-term performance of the prosthesis.

Currently, the so far used transcatheter TEHVs are characterized by two major challenges: the use of xenogeneic cell sources and/or the absence of Valsalva sinuses.^9–22,27,31^ Xenogeneic cell sources (and, in particular, ovine cells) are still largely adopted by the scientific community for the development of TEM-based TEHVs.^9,16–18,20,22,24^ This, however, significantly limits the translational potential of TEHVs, as it may result in severe immune reactions in human recipients, as observed for decellularized xenogeneic prostheses.^32,33^ The use of human cells for TEM-based TEHVs manufacturing will considerably reduce the risks of immunological reaction. However, only few preclinical studies utilized human-derived cell sources for the development of TEHVs.^19,24^

The sinuses of Valsalva are an anatomical feature typical of native semilunar valves, which promotes blood vortex formation in diastole, allowing for a progressive and active closure of the leaflets and preventing the build up of abnormal stresses in the leaflets.^34–36^ In this regard, we recently developed new sinus-stented tissue-engineered valves (TESVs) using ovine cell sources, which showed promising short-term performances in a technical feasibility study in a translational sheep model.^20^

In the present study, we propose a new culture protocol to produce clinically relevant off-the-shelf available valves with integrated Valsalva sinuses and based on a human cell-derived TEM (hTEM), the hTESVs. Human neonatal dermal fibroblasts (hDF) represent a valid cell source for producing hTESVs, because of their easy accessibility, rapid growth, high matrix protein production, and remodeling genes expression, which have shown similarities to human pediatric valvular interstitial cells.^19,24,37,38^ In addition, to stimulate matrix production even further,^39^ hDF were cultured in the presence of different concentration of transforming growth factor-β1 (TGF-β1). After in vitro characterization of the hTEM, the most-promising protocol was selected to produce decellularized hTESVs for TPVR. Thereafter, hTESVs functionality was assessed in vitro using a pulse duplicator system, followed by in vivo feasibility evaluation in a translational sheep model.

## Results

### Influence of TGF-β1 on hTEM development

**Fig. 1:**
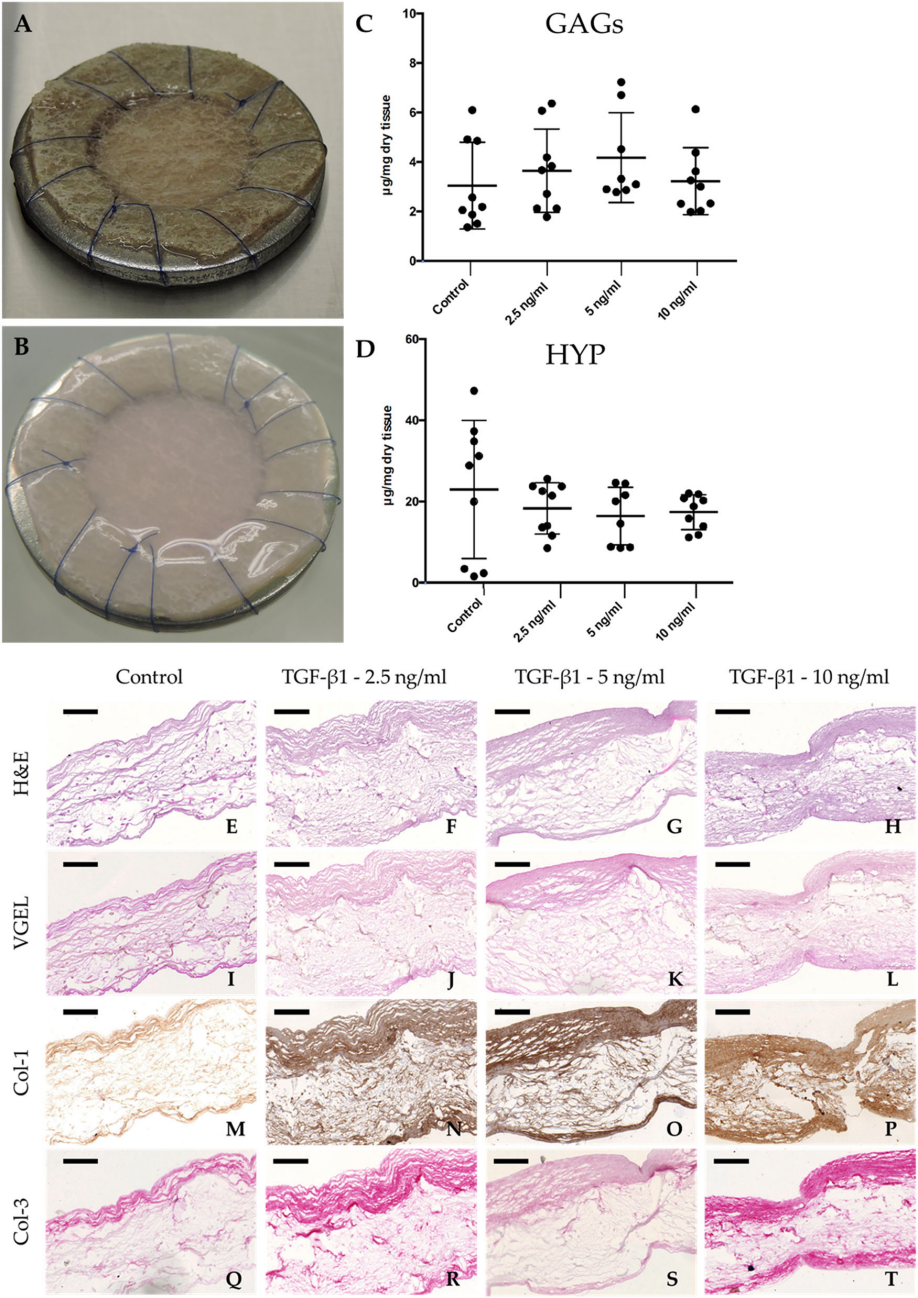
Qualitative evaluation of hTEM patches cultured with hDF and quantitative tissue composition after 4 weeks culture at different TGF-β1 concentrations. **a** Representative image of the polyglycolic acid/poly-4-hydroxybutyrate scaffold sutured onto a stainless metal ring before in vitro culture. **b** Representative image of a hTEM after 4 weeks culture showing shiny neo-tissue deposition and homogeneous matrix distribution. **c** GAGs content showed slightly higher amount in patches cultured at 5 ng/ml TGF-β1. **d** HYP content showed similar expression values among all the different groups. **e**–**h** Representative images of H&E staining show more pronounced neo-tissue formation for hTEM cultured with TGF-β1 in comparison with the control group. **i**–**l** VGEL staining did not show any expression of elastic fibers at 4 weeks follow-up. Col-1 **m**–**p** and Col-3 **q**–**t** deposition is enhanced in hTEM cultured in the presence of TGF-β1 (scale bars 100 μm). Dot plot graphs present mean ± standard deviation (center line and bounds of error bars, respectively)

After 4 weeks of culture, hTEM cultured in presence of TGF-β1 showed shiny neo-tissue formation and homogeneous extracellular matrix (ECM) deposition (Fig. 1a, b).

Quantitative analyses for the content of glycosaminoglycans (GAGs) and hydroxyproline (HYP) were performed for every hTEM culture condition after 4 weeks (Fig. 1c, d). GAG analysis showed comparable expression values between the control group (3.04 ± 1.75 µg/mg dry tissue) and the TGF-β1 supplemented groups (3.22 ± 1.35 µg/mg dry tissue, 4.17 ± 1.81 µg/mg dry tissue and 3.65 ± 1.68 µg/mg dry tissue for the 10 ng/ml, 5 ng/ml and 2.5 ng/ml TGF-β1 groups, respectively) (Fig. 1c, Supplementary Table 1). HYP analysis revealed no significant difference among the control group (22.96 ± 17.02 µg/mg dry tissue) and the TGF-β1 supplemented groups (17.36 ± 4.29 µg/mg dry tissue, 16.40 ± 7.10 µg/mg dry tissue, and 18.30 ± 6.34 µg/mg dry tissue for the 10 ng/ml, 5 ng/ml and 2.5 ng/ml TGF-β1 groups, respectively) (Fig. 1d, Supplementary Table 1).

Histological analysis was used to assess the ECM structure in the hTEM cultured with different TGF-β1 concentrations. Hematoxylin and Eosin (H&E) staining demonstrated a more compact and organized neo-tissue structure in the groups supplemented with TGF-β1, in comparison with the control group (Fig. 1e–h). Elastica Van Gieson (VGEL) staining (Fig. 1i–l) revealed the absence of elastic fibers, while Collagen 1 (Col-1) and Collagen 3 (Col-3) immuno-staining (Fig. 1m–t) confirmed the presence of a collagenous matrix rich in collagen type 1 and 3.

### Manufacturing of hTESVs

**Fig. 2:**
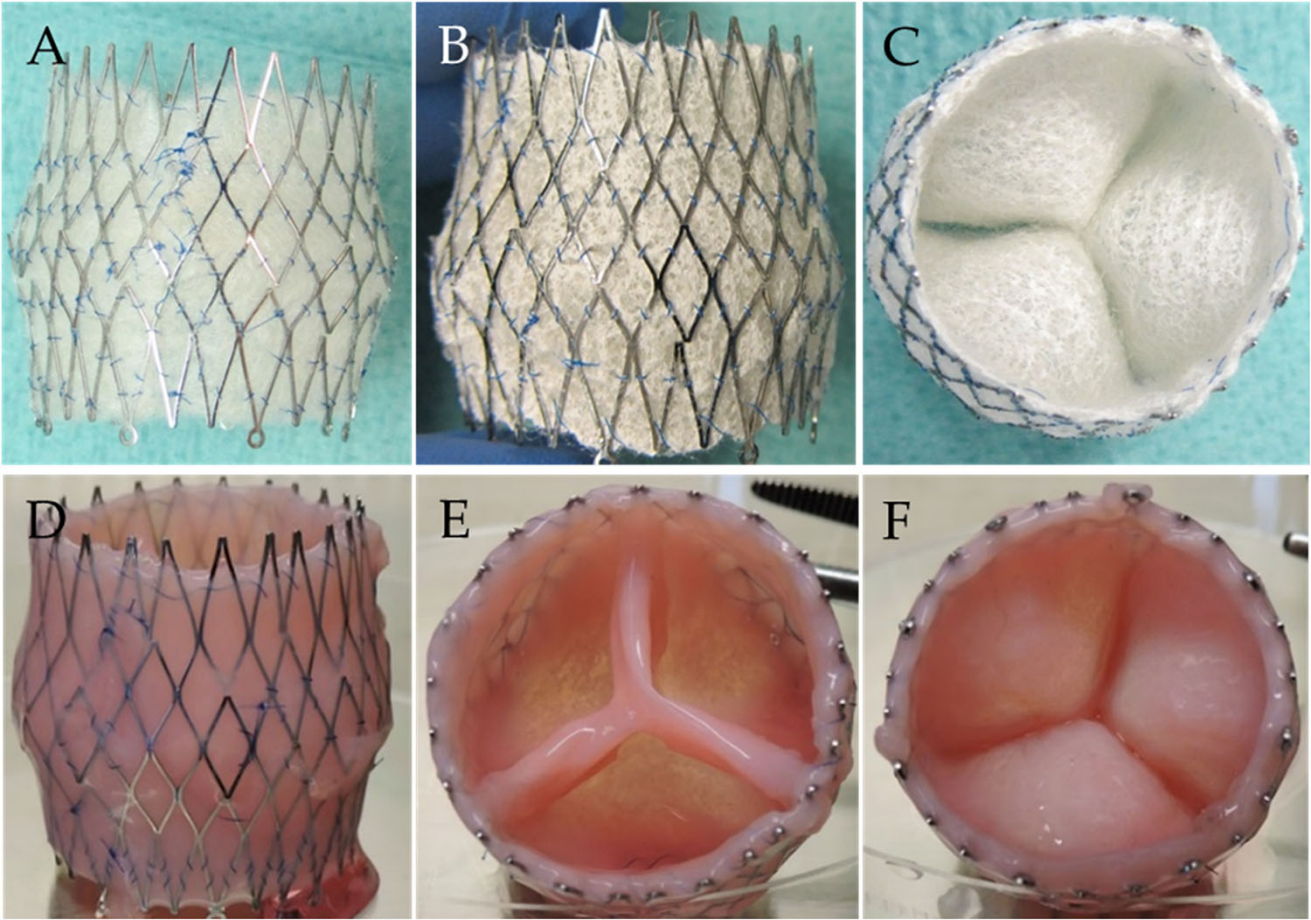
Manufacturing process of a hTESV. **a** PGA scaffold is sutured and integrated into the nitinol stent using continuous sutures. **b**, **c** PGA scaffold is coated with 1% P4HB in tetrahydrofuran. **d**–**e** hTESVs show dense, shiny, and homogeneous tissue formation laterally **d**, distally **e**, and proximally **f**, which was retained after decellularization procedure

Integration of the polyglycolic acid (PGA) scaffold within the sinus stent was successful, and the morphology of the wall and leaflets were retained after coating with poly-4-hydroxybutyrate (P4HB) (Fig. 2a–c). After 4 weeks of in vitro culture in the bioreactor with physical constraints, hTESVs (*n* = 10) showed dense, shiny, and homogeneous ECM formation with pliable leaflets (Fig. 2d–f), which was retained after the decellularization process.

### In vitro characterization of hTESVs

**Fig. 3:**
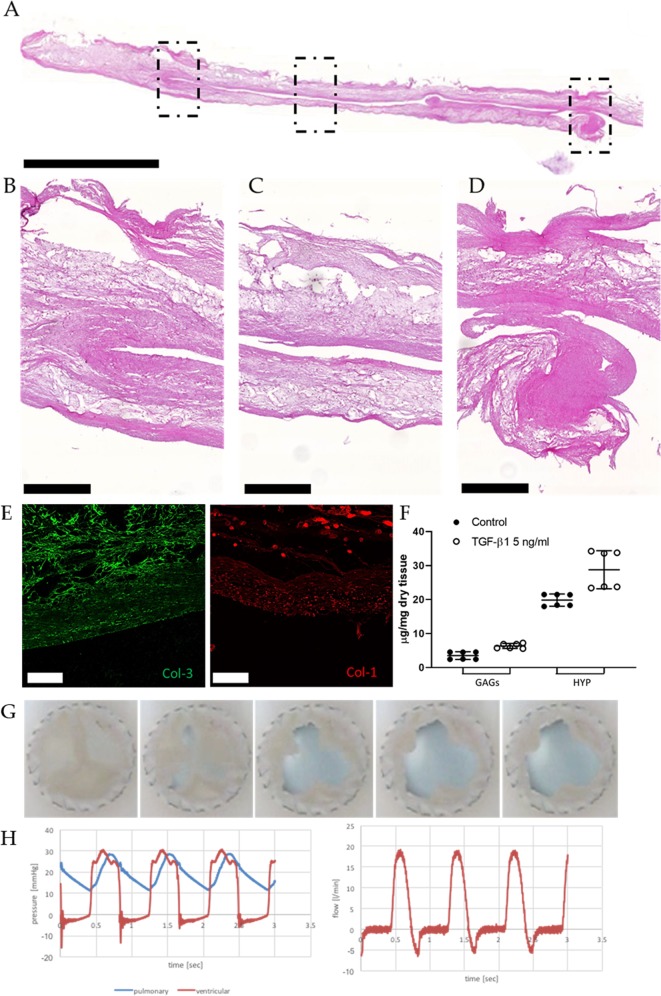
In vitro characterization of the hTESVs after 4 weeks culture. **a** H&E staining shows the general hTESV morphology comprising a dense collagenous matrix and the scaffold core (scale bar: 5000 μm). **b**–**d** Magnification pictures of the hinge region **b**, middle of the leaflet and wall **c**, and tip of the leaflet **d** marked by the dotted rectangles in **a** (scale bars: 500 μm). **e** Immunofluorescence staining shows the presence of Collagen 3 (green) and Collagen 1 (red) fibers in the newly formed matrix (scale bars: 100 μm). **f** Quantitative tissue composition demonstrates the slightly higher amount of GAGs and HYP in hTESV cultured in presence of 5 ng/ml TGF-β1 (white points) in comparison with control hTESV (black points). **g** Sequence of the opening and closing behavior of hTESVs tested in a valve pulse duplicator system for 1 hour at pulmonary pressure conditions. **h** Pressure and flow values for a representative hTESV after 1 hour in vitro valve testing; in the left panel, blue and red indicate pulmonary pressure and ventricular pressure, respectively; in the right panel, red indicates flow rate over time. Dot plot graphs present mean ± standard deviation (center line and bounds of error bars, respectively)

Histological H&E analysis confirmed the formation of a dense ECM on the PGA/P4HB scaffold and the absence of cell remnants after decellularization (Fig. 3a–d). Immunofluorescence analysis revealed the presence of both Col-3 and Col-1 (Fig. 3e). Quantitative biochemical analyses demonstrated an overall higher content of GAGs (6.35 ± 0.73 µg/ml) and HYP (28.78 ± 5.60 µg/ml) for TGF-β1 supplemented hTESVs compared to the untreated group (3.50 ± 1.13 µg/ml and 19.85 ± 1.78 µg/ml respectively for GAGs and HYP) (Fig. 3f).

Based on previously reported protocols,^13,18^ hTESVs were tested for in vitro functionality for up to 1 hour under physiologic pulmonary hemodynamic conditions (Fig. 3g, h). Valves did not show any sign of damage, tear, or deformation before in vitro test (Supplementary Fig. 1A, B), after crimping (Supplementary Fig. 1C, D), in the valve holder (Supplementary Fig. 1E, F), or after the test (Supplementary Fig. 1G, H). Opening and closing behavior of valve leaflets was normal, with full opening in systole, and complete closure during diastole. hTESVs (*n* = 2) demonstrated good performance for up to 1 hour at pulmonary pressure conditions, without signs of central nor paravalvular regurgitation and a peak systolic pressure of 27.85 ± 0.5 mmHg (Fig. 3g, h).

### In vivo performance of hTESVs and post mortem evaluation

**Fig. 4:**
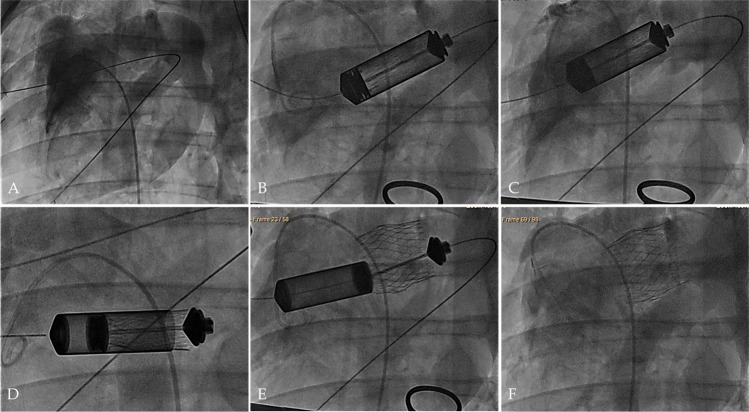
Transapical hTESV delivery under fluoroscopy guidance. **a** Imaging of the native pulmonary root. **b**, **c** Insertion and alignment of the delivery system to the implantation site. **d**, **e** Controlled positioning and deployment of the hTESV. **f** Final evaluation of the deployed hTESV confirming sufficient positioning and good valve functionality

**Fig. 5:**
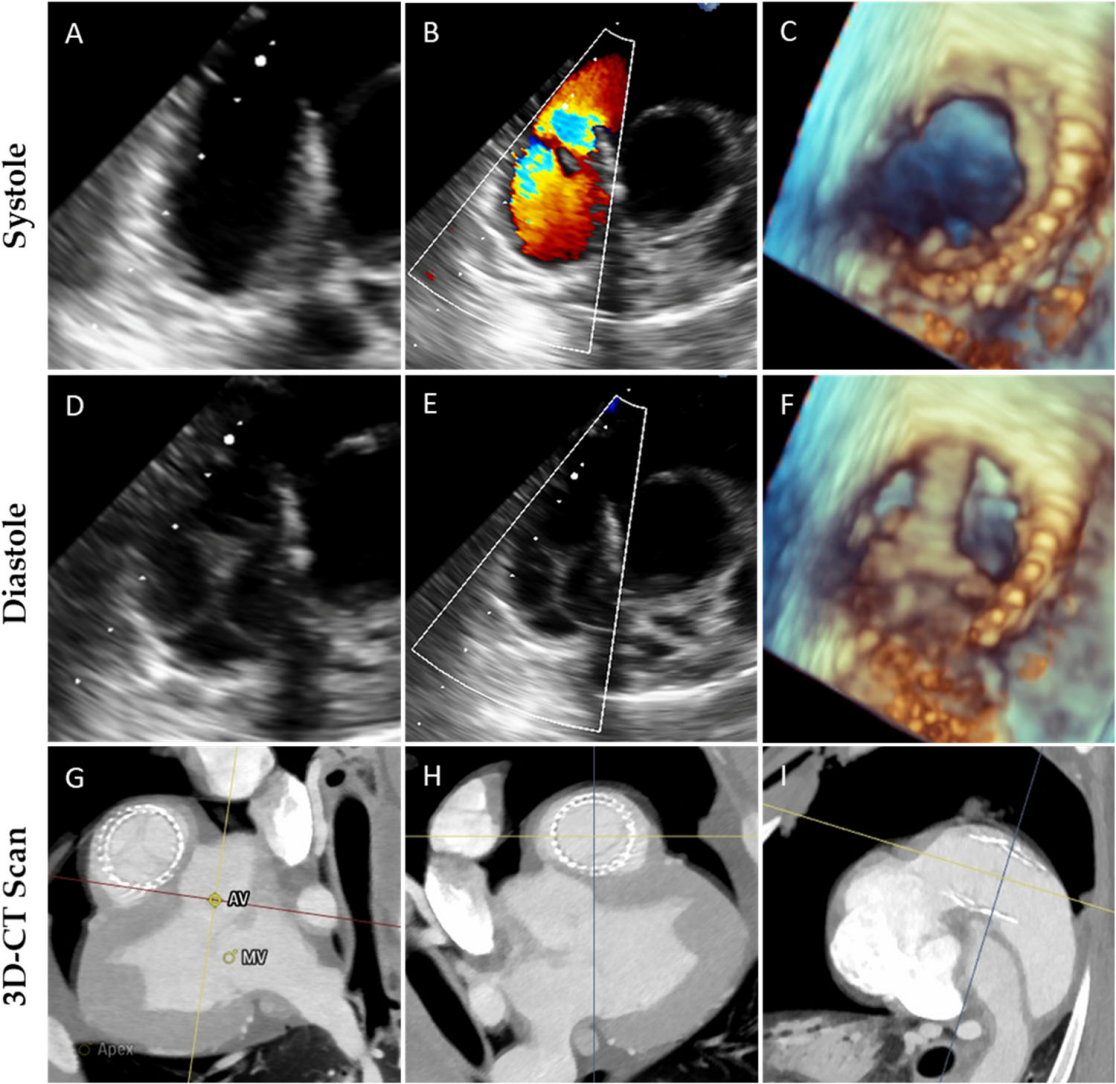
Echocardiographic assessment of valve functionality in vivo. **a**–**f** 2D images of hTESV show good leaflet motion during systole **a**–**c** and diastole **d**–**f**. **b**, **e** 2D-Doppler of hTESV demonstrates absence of anomalies in the forward flow, no regurgitation nor paravalvular leakage. **c**, **f** 3D representation of hTESV showing proper leaflet mobility during systole **c** and diastole **f**. **g**–**i** 3D-CT reconstruction performed pre-operatively shows the adult sheep heart and the valve implantation site from a proximal **g**, distal **h**, and lateral **i** perspective, confirming the correct positioning of the hTESV in the right ventricular outflow tract, over the native valve. AV = aortic valve, MV = mitral valve

To demonstrate the feasibility and safety of using hTESVs as pulmonary valve replacement, the hTESVs (*n* = 3) were crimped and loaded into the delivery device before successful transapical implantation. Contrast angiography confirmed sufficient valve positioning over the native pulmonary valve, and no evidence of central regurgitation (Fig. 4a–f, Supplementary Video 1). Bi- and three-dimensional (2D- and 3D)-echocardiographic assessment displayed good hTESV performance with complete coaptation and pliable leaflets (Fig. 5a–f). No paravalvular leakage, central regurgitation, or stenosis were detected, and leaflet motion was normal in all animals (Fig. 5a–f). No post-operative complications such as embolization or acute valve thrombosis occurred. After deployment, 3D computed tomography (3D-CT) reconstruction of the pulmonary root confirmed correct hTESV positioning (Fig. 5g–i). After the planned follow-up of up to 4 hours,^19,20^ the animals were euthanized and the hTESVs were harvested for post-mortem analysis.

**Fig. 6:**
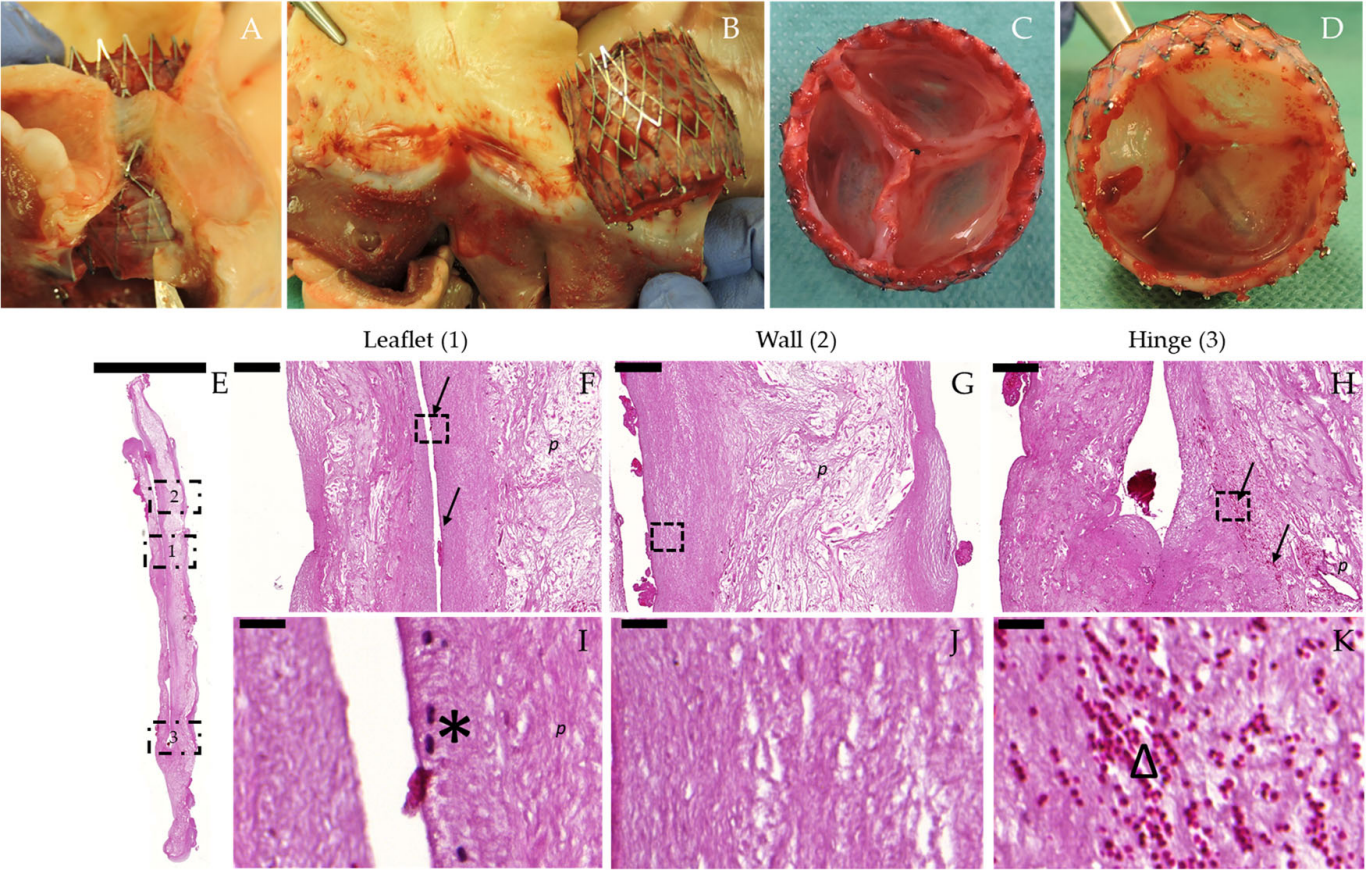
Post-mortem macroscopical and histological evaluation of hTESVs after 4 hours follow-up. All three hTESVs were correctly positioned at the entrance of the pulmonary root without signs of stent migration. Representative images of the H&E staining were performed on the valve cross-section. **a**, **b** Correct positioning of the hTESV confirmed by the presence of native leaflets entrapped in the stent struts. **c**, **d** hTESV showing thrombus-free smooth surfaces and absence of tears or ruptures. **e** H&E staining shows retained valve morphology after 4 hours in vivo follow-up (scale bar: 5000 μm). **f**–**h** H&E staining of the region marked by the dotted rectangles in **e** (1–3) shows host cellular infiltration predominantly at the surface and in the hinge region of the hTESVs (black arrows) (scale bars: 200 μm). **i**–**k** 60X magnification pictures of region marked by the dotted rectangles in **f**–**h** present an organized and packed collagenous matrix together with infiltrated leukocytes (black asterisks, **I**) and erythrocytes (triangle, **k**) (scale bars: 25 μm). **p** = polymer remnants

Post-mortem macroscopic evaluation further confirmed appropriate positioning of the hTESVs in the pulmonary root, without signs of stent migration, thrombus formation, leaflet tears, or rupture (Fig. 6a–d). No damages associated to crimping procedures were observed. Qualitative histological evaluations of the explanted hTESVs allowed to assess general valve morphology and cellular infiltration after 4 hours. Minimal fibrin deposition was observed on the hTESVs surface (Fig. 6a–e) and tissue structure was well preserved. Early host cellular infiltration was observed in proximity of the wall surface and in the hinge region of the hTESVs (Fig. 6f, h, black arrows) and was predominantly characterized by the presence of leukocytes (Fig. 6i, black asterisk) and erythrocytes (Fig. 6k, triangle).

## Discussion

Transcatheter heart valve substitutes are rapidly evolving as alternative treatment to surgical valve replacement.^5,40^ Nevertheless, current clinically available bioprostheses for TPVR suffer from major drawbacks such as continuous degeneration and structural deterioration, making them inadequate, especially for young adults and child patients. To address this increasing clinical need, TEHVs have been proposed as a potential solution, being characterized by properties such as long-term durability and regeneration capacities.^5,40^

Decellularized in vitro grown TEM-based valves have been successfully applied as TPVR in numerous preclinical studies in non-human primate and ovine models.^16–22^ Recently, their long-term durability, remodeling potential, and clinical relevance have been highlighted in a successful 1-year follow-up study.^18^ Nevertheless, xenogeneic cell sources have been often employed to produce the TEM, hampering the clinical translation of such prostheses. Recent investigations evaluated the functionality and remodeling capacities of non-fixed decellularized xenogeneic heart valves. After promising preclinical results,^41,42^ such prostheses have advanced into a clinical setting and were implanted in pediatric patients, resulting in severe immune reactions and fatal failure of the prostheses,^32^ thus raising many concerns on their safety.^33^ Therefore, the transition of culture protocols from animal-derived to human-derived cell sources is of pivotal importance for the safe translation of TEHVs into clinical practice.

In this context, the production of a robust human cell-derived TEM is fundamental to ensure immediate functionality of the TEHVs exposed to the hemodynamic loading. Here, we developed new culture protocols using hDF, to produce a compact and organized hTEM. Other groups previously investigated the potential of hDF for cardiovascular TE applications.^24,37,43^ These studies showed promising results in terms of functionality and host response, including recellularization and absence of calcifications.

Based on this promising data, we here further developed in vitro culture protocols for TEHVs with increased translational potential.

With the addition of TGF-β1, we stimulated collagen production and increased tissue organization in vitro.^44,45^ TGF-β1 is a well-known cytokine involved in several physiological processes like valvulogenesis,^46^ valve repair,^47^ cell proliferation, and ECM production.^44,45^ Our results suggested that the supplementation of 5 ng/ml of TGF-β1 led to increased GAGs production, comparable HYP content, and a qualitatively denser and more reproducible collagenous matrix in comparison with the other tested groups. Importantly, by extending the in vitro culture time, it will be possible to obtain a more robust matrix capable to sustain the aortic pressure conditions.^19^

Clinical translation of tissue-engineered products is challenged by many factors. To date, the large heterogeneity of culture methods reported in literature, the risks of infection during culture, the non-automated and non-scalable procedures, and the disregard of xeno-free protocols still limit the advancement of TEHVs to a clinical setting.^48,49^ However, although they are not yet clinically approved in the setting of heart valves, small caliber conduits produced with this approach, have already been advanced into clinical application providing safe and functional results.^40,50,51^ To hasten bench-to-bedside translation, it is necessary to establish Good Manufacturing and Good Laboratory Practices (GMP, GLP) procedures. The development of standardized manufacturing processes and quality control systems, both compliant with the International Organization for Standardization (ISO), have to be considered in order to satisfy the technical and infrastructural requirements for the commercialization of TEHVs.^48,52^

In addition, valve design has been recognized to be a key factor in order to achieve TEHVs long-term functionality and positive remodeling.^18,28–30^ In this regard, new computational modeling studies have recently underlined that physiological-like pre-shaping of TEHVs during in vitro culture may be a key player for the long-term durability and functionality of such substitutes.^18,28,29^ Here, we further enhanced this concept by introducing another fundamental anatomical feature of the native valves: the Valsalva sinuses.

The heart valve sinuses have the physiological role of actively promote and influence the opening and closing behavior of the leaflets, generate blood vortices, and consequently blood wash-out during diastole.^35,36^ Through this mechanism, the Valsalva sinuses are responsible for the decreased mechanical stress and strain sensed by the leaflets, thereby distributing the hemodynamic loading and preventing radial cusps contraction.^28,36,53–56^

From a clinical point of view, the importance of the Valsalva sinuses has been described in several surgical preservation and reconstruction procedures showing the benefits that such anatomical features bring to the patients.^57–59^ Despite this knowledge, clinically available transcatheter bioprostheses do not integrate Valsalva sinuses in their design.

Hence, based on a previously reported study from our group^18^ as well as others,^36,55,60,61^ we hypothesized that by implementing the Valsalva sinuses in our initial valve geometry, we could favor valve functionality and its future remodeling.

So far, only two studies have reported the implementation of Valsalva sinuses into TEHVs.^20,62^ Dodge–Khatami and colleagues^62^ used decellularized equine valved jugular veins as TPVR in sheep, showing promising functionality and endothelialization potential. However, the use of non-fixed xenogeneic material may represent a significant limitation for their future clinical translation.

In a recent proof-of-concept study, we demonstrated the technical feasibility of using sinus-stented-TEHVs as TPVR and showed their functionality in a short-term follow-up period (up to 16 weeks).^20^ However, owing to a suboptimal initial valve design, leaflet retraction and valve insufficiency occurred over time.

To overcome this limitation, in the present study, we have modified the valve design by (i) implementing physiological belly-shape in the leaflets, (ii) increasing the coaptation area, and (iii) using a different suturing pattern to integrate the scaffold into the stent prior to in vitro culture. In addition, the hTESVs described here, were manufactured with a human cell source, thereby enhancing their clinical relevance. This resulted in successful in vitro valve testing and in vivo functionality, with optimal valve performance up to 4 hours follow-up in a translational FDA-recommended ovine model without evidence of regurgitation or stenosis. Chronic studies are however needed to confirm the remodeling potential of the newly developed hTEM as well as the long-term functionality of the prosthesis.

Our study has several limitations. First, the number of animals used in this study was limited, but sufficient to prove the feasibility of our approach. Second, short- (e.g., 1–3 months) and long-term (≥6 months) follow-up are fundamental in order to assess valve functionality and remodeling over time, even if the remodeling potential of a similar matrix was previously confirmed by others.^23,24^ Compared with our previous work,^20^ major changes (i.e., different cell source, different valve and leaflet geometry) were applied to obtain the hTESV and, therefore, the novel valve design needed to be tested in an acute feasibility study. Although chronic experiments were beyond the scope of this study, they should be addressed in the future. Third, the stress distribution on the leaflets should be validated with computational modeling tools, whereas the flow patterns determined by the implemented Valsalva sinuses should be confirmed using flow magnetic resonance imaging technologies.

In conclusion, in this study we demonstrated the possibility to develop a human cell-derived TEM for heart valve tissue engineering applications. In addition, we proposed a novel valve design, which includes the anatomical feature of the Valsalva sinuses, and we showed the feasibility and safety of TPVR using an off-the-shelf available human cell-derived TESVs in an ovine model.

## Methods

### In vitro manufacturing and culture of hTEM

Scaffold patches (*n* = 36, triplicates) (area = 6 cm^2^) were produced using non-woven PGA meshes (Confluent) and coated with 1% P4HB (TEPHA Inc.) in liquid tetrahydrofuran (Sigma-Aldrich). Scaffolds were then sutured onto stainless steel rings (Hasler) and sterilized in ethanol following previously reported protocols.^63^

Neonatal hDF (from three different donors, mycoplasma free) were purchased from CellSystems Biotechnologie GmbH and expanded in advanced Dulbecco’s Modified Eagle Medium (Sigma-Aldrich) supplemented with 10% fetal bovine serum (Invitrogen AG), 1% Glutamax (Invitrogen AG), and 1% Penicillin–Streptomycin (Invitrogen AG), and cultured in a cell incubator at 37 °C. Afterwards, cells (1.0 × 10^6^ cells/cm^2^) were seeded onto the patches using fibrin as cell carrier.^64^

After seeding, patches were cultured at 37 °C on an orbital shaker. Tissue culture medium was supplemented with l-ascorbic acid two-phosphate (0.25 mg/ml; Sigma-Aldrich) and medium was replaced every 2–3 days. In addition, to investigate the role of TGF-β1 on ECM production, patches were also supplemented with different concentrations of TGF-β1 (Peprotech): control (0 ng/ml TGF-β1), 2.5 ng/ml TGF-β1, 5 ng/ml TGF-β1, and 10 ng/ml TGF-β1 (*n* = 9 per group). After 4 weeks of culture, hTEM were decellularized by using a detergent solution (0.25% Triton X-100, sodium deoxycholate and 0.02% ethylenediaminetetraacetic acid) followed by a Benzonase treatment (EMD Millipore), as previously described.^16^

### In vitro tissue analyses of hTEM

Quantification of GAGs and HYP (as an indicator for collagen content) amounts were performed, as previously described,^65,66^ for every hTEM-patch cultured at the different TGF-β1 conditions (*n* = 36). In brief, samples were digested using a buffer solution containing 6 mm papain (Sigma-Aldrich) at 60 °C overnight. Subsequently, GAGs amount was compared with shark cartilage chondroitin sulfate (10 mg/ml, Sigma-Aldrich) as previously described.^65^ Finally, HYP amount was compared with a HYP standard solution (10 mg/ml, Sigma-Aldrich) following a previously established protocol.^66^ All samples were reproduced as triplicates. All concentrations were normalized to the total dry weight of the samples.

Representative hTEM were analyzed qualitatively for matrix composition using histology and immunohistochemistry (Ventana Benchmark) techniques. Briefly, hTEM were formalin-fixed, paraffin-embedded, and cut consecutively into 5 µm slices. The tissue sections were analyzed using H&E, Col-1 (Abcam, ab34710, 1:200), Col-3 (Abcam, ab7778, 1:250), and VGEL staining. The stained samples were imaged with brightfield microscopy (Mirax Midi Microscope, Carl Zeiss GmbH) and analyzed with Pannoramic Viewer software (3DHISTECH).

### Manufacturing and in vitro culture of hTESVs

Trileaflet sinus-shaped heart valve scaffolds (*n* = 10) were fabricated by adapting a previously described protocol,^17^ using non-woven PGA scaffolds, sewn to radially self-expandable sinus-shaped nitinol stents (stent diameter: 28 mm distal, 35 mm mid-part, and 30 mm proximal; CARAG AG) by using continuous sutures (Yavo, polyvinylidene difluoride, non-resorbable, 6/0 USP). Previously developed TESVs^20^ were grown onto a straight nitinol stent and then carefully readapted and sewn onto a nitinol sinus stent. Here, we developed a novel scaffold design by creating custom-made molds to obtain valve walls and leaflets that are compatible with the sinus stent. Specifically, the new lower walls were specifically designed to fit the sinus-stent profile, and leaflet length was increased to provide a longer coaptation area, as previously described.^18^ Thereafter, the scaffold was coated with 1% P4HB (TEPHA Inc.) in liquid tetrahydrofuran (Sigma-Aldrich), dried overnight, and sterilized in ethanol, following previously reported protocols.^63^

After hDF expansion in medium, 1.0 × 10^6^ cells/cm^2^ were seeded onto the sinus-stented heart valve scaffolds using fibrin as cell carrier.^64^ After seeding, tissue culture inserts were introduced as physical constraints for the leaflets and the valves were then cultured into the Diastolic Pulse Duplicator bioreactor system for 4 weeks.^63^ The valve housings of the bioreactor were custom-designed to fit the size of the sinus stent. Medium was supplemented with l-ascorbic acid 2-phosphate (0.25 mg/ml; Sigma-Aldrich) and TGF-β1 (5 ng/ml, Peprotech). An additional control valve was cultured without the addition of TGF-β1. Medium was replaced every 2–3 days. After 4 weeks of culturing, hTESVs were decellularized as previously described.^16^

### In vitro tissue analyses and in vitro performance of hTESVs

GAGs and HYP contents were analyzed for hTESVs cultured with and without TGF-β1 (*n* = 1 for both conditions) as previously described for hTEM.^65^

hTESVs were analyzed qualitatively for matrix composition using histology (H&E) and immunofluorescence. Samples of the leaflets were fixed in formalin and processed as previously described for hTEM.

Immunofluorescence was utilized to confirm the presence of Col-1 and Col-3 proteins on a non-implanted valve. In brief, hTESVs sections were formalin-fixed, paraffin-embedded, and cut consecutively into 5 µm slices. Primary antibodies for Col-1 (Abcam, ab23446, 1:200) and Col-3 (Abcam, ab7778, 1:250) were applied followed by the relative secondary antibodies (Lifetechnologies, Thermo Fisher Scientific, A11004 and A11008 for Col-1 and Col-3, respectively, 1:400). The stained sections were visualized using a fluorescence slide scanner (ZEISS Axio Scan.Z1, Carl Zeiss AG). Image processing was performed using the Leica Las X Life Science imaging software (Leica Microsystems).

hTESVs functionality was tested and visualized in a hydrodynamic pulsatile test system (HDT-500, BDC Laboratories) as previously described,^29^ using a high-speed camera (MotionScope M-5). For this purpose, a newly developed customized silicon annulus mold comprising apposite pockets for the sinus-shaped stent was used as valve holder. hTESVs cultured with TGF-β1 (*n* = 2) and without TGF-β1 (*n* = 1) were exposed to physiologic pulmonary pressure conditions for 1 hour (peak systolic pressure of 27 mmHg; end-diastolic pressure of 10 mmHg; 72 bpm). Measures for peak systolic pressure and regurgitation fraction were obtained for each valve after 10 minutes and 1 hour of tests.

### Delivery system and crimping procedure

A custom-made hydraulic delivery system was designed and manufactured by CARAG AG to enable transapical delivery of hTESVs. The system comprised a 14 mm-stainless steel capsule (valve chamber) to accommodate the valve, and a flexible delivery catheter.^20^ Previously developed crimping device and tools^20^ were used to allow the crimping and insertion of the hTESVs into the valve chamber. Delivery tests were performed on all hTESVs (*n* = 3) prior implantation.

### In vivo transcatheter implantation of hTESVs

The animal study was conducted at the Division of Surgical Research based at the University Hospital of Zurich, and all animals received humane care in accordance with the “Principles of laboratory animal-care” and the “Guide for the care and use of laboratory animals” of the National Institutes of Health. All the procedures received the approval of the Cantonal Veterinary Office (License number ZH_041_2017) and performed in accordance with the European Union guidelines (86/609/EEC) and Swiss Federal animal protection law and ordinance.

To evaluate the feasibility and safety, the acute performance of hTESVs as TPVR was tested in three sheep (female white alpine sheep, 50–55 kg) selected based on their annulus size (25.9 ± 0.2 mm at peak systolic pressure). The animals underwent surgery through anterolateral–thoracic access and antegrade approach at the third intercostal space. After mini-thoracotomy and pericardiotomy, the right ventricular apex was punctured with two purse-string sutures, and the delivery system was inserted under fluoroscopy (ALLURA FD 20/20., Philips Electronics). A baseline contrast angiography was performed to assess the native pulmonary valve and root. After correct positioning of the device, the valve was released under fluoroscopy guidance.

Planned follow-up for all animals (*n* = 3) was up to 4 hours after implantation. Transesophageal echocardiography (TEE; Philips Healthcare iE33W xMATRIX Ultrasound) and angiography were performed immediately after deployment and before euthanization, to assess hTESVs functionality. The study did not include randomization or blind investigator.

### Valve positioning and acute functionality

2D- and 3D-TEE were performed immediately after implantation (*n* = 3) to evaluate the performance of the hTESVs. The following parameters were determined: orifice diameter, degree of regurgitation, and leaflets motion. To confirm sufficient hTESV positioning, the pulmonary root was imaged and reconstructed using 3D-CT (Siemens) analysis, before and after implantation (*n* = 1).

### Post-mortem evaluation

After euthanization, the heart was harvested and the hTESVs (*n* = 3) were macroscopically examined for tissue integrity and then processed using histological analysis (Ventana Benchmark) to assess matrix composition and cellular infiltration. Samples of the leaflets were fixed in formalin and processed as previously described for hTEM and hTESVs. The tissue sections were analyzed using H&E staining.

### Statistics

GAGs, and HYP content quantification are expressed as mean ± standard deviation, obtained by averaging the mean value of each patch for each group. To compare the groups, unpaired non-parametric Kruskal-Wallis test with Dunn’s correction was performed, considering the non-Gaussian distribution of the data. *p* < 0.05 was considered as statistically significant. Statistics was elaborated with Prism software (v. 7, GraphPad).

### Reporting summary

Further information on research design is available in the Nature Research Reporting Summary linked to this article.

## Supplementary information

Supplementary Movie 1

Supplementary Information

Reporting Summary Checklist

## Data Availability

Data are available upon request and completion of a Materials Transfer Agreement (MTA).

## Supplementary information

**Supplementary Information** accompanies the paper on the *npj Regenerative Medicine* website (10.1038/s41536-019-0077-4).

## References

[1] Alkashkari W, Alsubei A, Hijazi ZM (2018). Transcatheter pulmonary valve replacement: current state of art. Curr. Cardiol. Rep..

[2] Ghawi H, Kenny D, Hijazi ZM (2012). Transcatheter pulmonary valve replacement. Cardiol. Ther..

[3] Kenny DP, Hijazi ZM (2017). Current status and future potential of transcatheter interventions in congenital heart disease. Circ. Res..

[4] Sarikouch S (2016). Decellularized fresh homografts for pulmonary valve replacement: a decade of clinical experience. Eur. J. Cardio-Thorac. Surg..

[5] Fioretta ES, Dijkman PE, Emmert MY, Hoerstrup SP (2017). The future of heart valve replacement: recent developments and translational challenges for heart valve tissue engineering. J. tissue Eng. Regen. Med..

[6] Wissing TB, Bonito V, Bouten CVC, Smits AIPM (2017). Biomaterial-driven in situ cardiovascular tissue engineering—a multi-disciplinary perspective. NPJ Regen. Med..

[7] Langer R, Vacanti JP (1993). Tissue engineering. Science.

[8] Cheung DY, Duan B, Butcher JT (2015). Current progress in tissue engineering of heart valves: multiscale problems, multiscale solutions. Expert Opin. Biol. Ther..

[9] Schmidt D (2010). Minimally-invasive implantation of living tissue engineered heart valves: a comprehensive approach from autologous vascular cells to stem cells. J. Am. Coll. Cardiol..

[10] Emmert MY (2011). Transapical aortic implantation of autologous marrow stromal cell-based tissue-engineered heart valves: first experiences in the systemic circulation. JACC Cardiovasc. Interv..

[11] Emmert MY (2014). Transcatheter aortic valve implantation using anatomically oriented, marrow stromal cell-based, stented, tissue-engineered heart valves: technical considerations and implications for translational cell-based heart valve concepts. Eur. J. Cardio-Thorac. Surg..

[12] Weber B (2011). Injectable living marrow stromal cell-based autologous tissue engineered heart valves: first experiences with a one-step intervention in primates. Eur. Heart J..

[13] Kluin J (2017). In situ heart valve tissue engineering using a bioresorbable elastomeric implant - from material design to 12 months follow-up in sheep. Biomaterials.

[14] Serruys PW (2017). Restorative valve therapy by endogenous tissue restoration: tomorrow’s world? Reflection on the EuroPCR 2017 session on endogenous tissue restoration. EuroIntervention.

[15] Bennink G (2018). A novel restorative pulmonary valved conduit in a chronic sheep model: Mid-term hemodynamic function and histologic assessment. J. Thorac. Cardiovasc. Surg..

[16] Dijkman PE, Driessen-Mol A, Frese L, Hoerstrup SP, Baaijens FP (2012). Decellularized homologous tissue-engineered heart valves as off-the-shelf alternatives to xeno- and homografts. Biomaterials.

[17] Driessen-Mol A (2014). Transcatheter implantation of homologous “off-the-shelf” tissue-engineered heart valves with self-repair capacity: long-term functionality and rapid in vivo remodeling in sheep. J. Am. Coll. Cardiol..

[18] Emmert Maximilian Y., Schmitt Boris A., Loerakker Sandra, Sanders Bart, Spriestersbach Hendrik, Fioretta Emanuela S., Bruder Leon, Brakmann Kerstin, Motta Sarah E., Lintas Valentina, Dijkman Petra E., Frese Laura, Berger Felix, Baaijens Frank P. T., Hoerstrup Simon P. (2018). Computational modeling guides tissue-engineered heart valve design for long-term in vivo performance in a translational sheep model. Science Translational Medicine.

[19] Lintas V (2018). Development of a novel human cell-derived tissue-engineered heart valve for transcatheter aortic valve replacement: an in vitro and in vivo feasibility study. J. Cardiovasc. Transl. Res..

[20] Motta SE (2018). Development of an off-the-shelf tissue-engineered sinus valve for transcatheter pulmonary valve replacement: a proof-of-concept study. J. Cardiovasc. Transl. Res..

[21] Schmitt B (2016). Percutaneous pulmonary valve replacement using completely tissue-engineered off-the-shelf heart valves: six-month in vivo functionality and matrix remodelling in sheep. EuroIntervention.

[22] Weber B (2013). Off-the-shelf human decellularized tissue-engineered heart valves in a non-human primate model. Biomaterials.

[23] Reimer J (2017). Implantation of a tissue-engineered tubular heart valve in growing lambs. Ann. Biomed. Eng..

[24] Syedain Z (2015). 6-month aortic valve implantation of an off-the-shelf tissue-engineered valve in sheep. Biomaterials.

[25] Flanagan TC (2009). In vivo remodeling and structural characterization of fibrin-based tissue-engineered heart valves in the adult sheep model. Tissue Eng. Part A.

[26] Gottlieb D (2010). In vivo monitoring of function of autologous engineered pulmonary valve. J. Thorac. Cardiovasc. Surg..

[27] Capulli AK (2017). JetValve: Rapid manufacturing of biohybrid scaffolds for biomimetic heart valve replacement. Biomaterials.

[28] Loerakker S, Ristori T, Baaijens FPT (2016). A computational analysis of cell-mediated compaction and collagen remodeling in tissue-engineered heart valves. J. Mech. Behav. Biomed. Mater..

[29] Sanders B (2016). Improved geometry of decellularized tissue engineered heart valves to prevent leaflet retraction. Ann. Biomed. Eng..

[30] Loerakker S, Argento G, Oomens CW, Baaijens FP (2013). Effects of valve geometry and tissue anisotropy on the radial stretch and coaptation area of tissue-engineered heart valves. J. Biomech..

[31] Emmert MY (2012). Stem cell-based transcatheter aortic valve implantation: first experiences in a pre-clinical model. JACC Cardiovasc. Interv..

[32] Simon P (2003). Early failure of the tissue engineered porcine heart valve SYNERGRAFT in pediatric patients. Eur. J. Cardio-Thorac. Surg..

[33] Namiri M (2017). Engineering natural heart valves: possibilities and challenges. J. tissue Eng. Regen. Med..

[34] Gharib M, Kremers D, Koochesfahani M, Kemp M (2002). Leonardo’s vision of flow visualization. Exp. Fluids.

[35] Salica A (2016). The combined role of sinuses of Valsalva and flow pulsatility improves energy loss of the aortic valve. Eur. J. Cardio-Thorac. Surg..

[36] Katayama S, Umetani N, Sugiura S, Hisada T (2008). The sinus of Valsalva relieves abnormal stress on aortic valve leaflets by facilitating smooth closure. J. Thorac. Cardiovasc. Surg..

[37] Syedain ZH, Bradee AR, Kren S, Taylor DA, Tranquillo RT (2013). Decellularized tissue-engineered heart valve leaflets with recellularization potential. Tissue Eng. Part A.

[38] Fahrenholtz MM (2014). Characterization of dermal fibroblasts as a cell source for pediatric tissue engineered heart valves. J. Cardiovasc. Dev. Dis..

[39] Clark RA, McCoy GA, Folkvord JM, McPherson JM (1997). TGF-beta 1 stimulates cultured human fibroblasts to proliferate and produce tissue-like fibroplasia: a fibronectin matrix-dependent event. J. Cell. Physiol..

[40] Motta SE, Lintas V, Fioretta ES, Hoerstrup SP, Emmert MY (2018). Off-the-shelf tissue engineered heart valves for in situ regeneration: current state, challenges and future directions. Expert Rev. Med. Devices.

[41] Dohmen PM (2012). Clinical results of implanted tissue engineered heart valves. HSR Proc. Intensive Care Cardiovasc. Anesth..

[42] Gallo M (2016). Decellularized aortic conduits: could their cryopreservation affect post-implantation outcomes? A morpho-functional study on porcine homografts. Heart Vessels.

[43] Syedain Zeeshan H., Graham Melanie L., Dunn Ty B., O’Brien Timothy, Johnson Sandra L., Schumacher Robert J., Tranquillo Robert T. (2017). A completely biological “off-the-shelf” arteriovenous graft that recellularizes in baboons. Science Translational Medicine.

[44] Gessin JC, Brown LJ, Gordon JS, Berg RA (1993). Regulation of collagen synthesis in human dermal fibroblasts in contracted collagen gels by ascorbic acid, growth factors, and inhibitors of lipid peroxidation. Exp. cell Res..

[45] Narine K (2004). Transforming growth factor-beta-induced transition of fibroblasts: a model for myofibroblast procurement in tissue valve engineering. J. Heart Valve Dis..

[46] Chester AH (2014). The living aortic valve: from molecules to function. Glob. Cardiol. Sci. Pract..

[47] Liu AC, Gotlieb AI (2008). Transforming growth factor-β regulates in vitro heart valve repair by activated valve interstitial cells. Am. J. Pathol..

[48] Emmert MY, Hoerstrup SP (2016). Tissue engineered heart valves: moving towards clinical translation. Expert Rev. Med. Devices.

[49] Fioretta ES, Dijkman PE, Emmert MY, Hoerstrup SP (2018). The future of heart valve replacement: recent developments and translational challenges for heart valve tissue engineering. J. Tissue Eng. Regen. Med..

[50] Lawson JH (2016). Bioengineered human acellular vessels for dialysis access in patients with end-stage renal disease: two phase 2 single-arm trials. Lancet.

[51] Kirkton RD (2019). Bioengineered human acellular vessels recellularize and evolve into living blood vessels after human implantation. Sci. Transl. Med..

[52] Fioretta E.S., von Boehmer L., Motta S.E., Lintas V., Hoerstrup S.P., Emmert M.Y. (2019). Cardiovascular tissue engineering: From basic science to clinical application. Experimental Gerontology.

[53] Pisani G (2013). Role of the sinuses of Valsalva on the opening of the aortic valve. J. Thorac. Cardiovasc Surg..

[54] Toninato R, Salmon J, Susin FM, Ducci A, Burriesci G (2016). Physiological vortices in the sinuses of Valsalva: an in vitro approach for bio-prosthetic valves. J. Biomech..

[55] Grande-Allen KJ, Cochran RP, Reinhall PG, Kunzelman KS (2000). Re-creation of sinuses is important for sparing the aortic valve: a finite element study. J. Thorac. Cardiovasc. Surg..

[56] Bottio T (2012). Aortic valve hydrodynamics: considerations on the absence of sinuses of Valsalva. J. Heart Valve Dis..

[57] Miller DC (2003). Valve-sparing aortic root replacement in patients with the Marfan syndrome. J. Thorac. Cardiovasc Surg..

[58] Al-Radi OO (2016). Aortic ring autograft for reconstruction of the neo-pulmonary root in the arterial switch operation. J. Thorac. Cardiovasc Surg..

[59] Settepani F (2009). Reimplantation valve-sparing aortic root replacement with the Valsalva graft: what have we learnt after 100 cases?. Interact. Cardiovasc. Thorac. Surg..

[60] Maselli D (2014). Differences in aortic cusp coaptation between the reimplantation and the remodeling techniques of aortic valve–sparing surgery: An in vitro porcine model study. J. Thorac. Cardiovasc. Surg..

[61] Thubrikar MJ, Nolan SP, Aouad J, Deck JD (1986). Stress sharing between the sinus and leaflets of canine aortic valve. Ann. Thorac. Surg..

[62] Dodge-Khatami A, Hallhagen S, Limacher K, Soderberg B, Jenni R (2012). Minimally invasive insertion of an equine stented pulmonary valve with a built-in sinus portion in a sheep model. Catheter. Cardiovasc. Interv..

[63] Mol A (2005). Tissue engineering of human heart valve leaflets: a novel bioreactor for a strain-based conditioning approach. Ann. Biomed. Eng..

[64] Mol A (2005). Fibrin as a cell carrier in cardiovascular tissue engineering applications. Biomaterials.

[65] Farndale RW, Buttle DJ, Barrett AJ (1986). Improved quantitation and discrimination of sulphated glycosaminoglycans by use of dimethylmethylene blue. Biochim. Biophys. acta.

[66] Huszar G, Maiocco J, Naftolin F (1980). Monitoring of collagen and collagen fragments in chromatography of protein mixtures. Anal. Biochem..

