# Supplementary material for: Human cell-derived tissue-engineered heart valve with integrated Valsalva sinuses: towards native-like transcatheter pulmonary valve replacements
Source: NPJ Regen Med. 2019 Jun 17;4:14. doi: 10.1038/s41536-019-0077-4 (PMC6572861; doi:10.1038/s41536-019-0077-4)
Supplement: Supplementary file 2 — Supplementary Information [file 41536_2019_77_MOESM2_ESM.pdf]

Human Cell-derived Tissue-engineered Heart Valve with Integrated Valsalva Sinuses: Towards Native-like Transcatheter Pulmonary Valve Replacements

Authors: Sarah E. Motta[1], Valentina Lintas[1], Emanuela S. Fioretta[1], Petra E. Dijkman[1], Matilde Putti[2], Etem Caliskan[3,4], Hector Rodriguez[3,4], Miriam Lipiski[5], Mareike Sauer[5], Nikola Cesarovic[5], Simon P. Hoerstrup[1,6], Maximilian Y. Emmert[1,3,4,6]

[1]*Institute for Regenerative Medicine (IREM), University of Zurich, Zurich, Switzerland*

[2]*Department of Biomedical Engineering, Technische Universiteit Eindhoven, The Netherlands*

[3]*Department of Cardiovascular Surgery, Charité Universitätsmedizin Berlin, Berlin, Germany*

[4]*Department of Cardiothoracic and Vascular Surgery, German Heart Center Berlin, Berlin, Germany*

[5]*Division of Surgical Research, University Hospital Zürich, University Zürich*

[6]*Wyss Translational Center Zurich, University and ETH Zurich, Zurich, Switzerland*

Running title: Human cell-derived tissue-engineered sinus valves

Address for correspondence

Prof. Maximilian Y. Emmert, MD, PhD

Institute for Regenerative Medicine (IREM)

University of Zurich

Wagistrasse 12

8952 Schlieren, Switzerland

Email: *Maximilian.Emmert@irem.uzh.ch*

Tel.: +41 44 634 56 10

Supplementary Information

Supplementary Table

Supplementary Table 1: Mean and standard deviation of HYP and GAG content obtained for the different hTEM patches cultured with distinct TGF-β1 concentrations.

	**Control**	**2.5 ng/ml TGF-β1**	**5 ng/ml TGF-β1**	**10 ng/ml TGF-β1**
**HYP** [μg/mg dry tissue]	22.96 ± 17.02	18.30 ± 6.34	16.40 ± 7.10	17.36 ± 4.29
**GAG** [μg/mg dry tissue]	3.04 ± 1.75	3.65 ± 1.68	4.17 ± 1.81	3.22 ± 1.35

Supplementary Figure

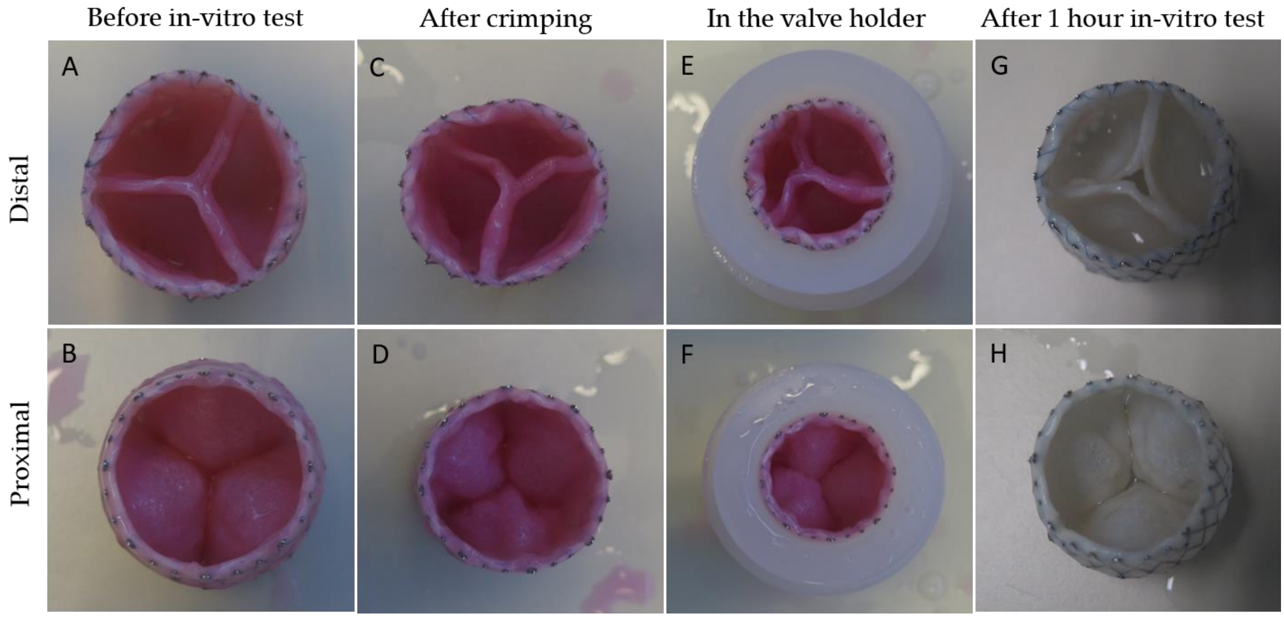

Supplementary Figure 1: Macroscopical appearance of the hTESVs during the in-vitro valve test. hTESV morphology, ECM integrity, and leaflet mobility were retained for every tested condition, that is before in-vitro test (A-B), after crimping (C-D), in the valve holder (E-F), and after 1-hour in-vitro tests (G-H).

Supplementary Movie

Supplementary Movie 1: Fluoroscopy-guided transapical delivery of a hTESV into pulmonary valve position.
